# Novel 3D-Printed Dressings of Chitosan–Vanillin-Modified Chitosan Blends Loaded with Fluticasone Propionate for Treatment of Atopic Dermatitis

**DOI:** 10.3390/pharmaceutics14091966

**Published:** 2022-09-18

**Authors:** Georgia Michailidou, Dimitrios N. Bikiaris

**Affiliations:** Laboratory of Polymer Chemistry and Technology, Department of Chemistry, Aristotle University of Thessaloniki, Thessaloniki 54124, Greece

**Keywords:** polymer blends, 3D printing, drug release, chitosan, fluticasone propionate, atopic dermatitis

## Abstract

In the present study, the blends of CS and Vanillin–CS derivative (VACS) were utilized for the preparation of printable inks for their application in three-dimensional (3D) printing procedures. Despite the synergic interaction between the blends, the addition of ι-carrageenan (iCR) as a thickening agent was mandatory. Their viscosity analysis was conducted for the evaluation of the optimum CS/VACS ratio. The shear thinning behavior along with the effect of the temperature on viscosity values were evident. Further characterization of the 3D-printed structures was conducted. The effect of the CS/VACS ratio was established through swelling and contact angle measurements. An increasing amount of VACS resulted in lower swelling ability along with higher hydrophobicity. Fluticasone propionate (FLU), a crystalline synthetic corticosteroid, was loaded into the CS/VACS samples. The drug was loaded in its amorphous state, and consequently, its in vitro release was significantly enhanced. An initial burst release, followed by a sustained release profile, was observed.

## 1. Introduction

Three-dimensional (3D) printing is a powerful technique vastly utilized for tissue engineering applications [1]. Currently, 3D printing is also applied in drug delivery systems since drug release can be modified by changing the infill percentage or geometry during printing [2]. An interesting application for 3D printing is the formation of patches for topical and transdermal applications, for the prolonged and sustainable delivery of the drugs. Over the years, various techniques have been utilized for the preparation of patches, including solvent casting [3], freeze-drying [4], and electrospinning [5], while the main key properties of their design are breathability, flexibility, good mechanical properties, and biocompatibility [6].

Over the years, various synthetic and natural polymers have been applied in the 3D bioprinting technique, including polycaprolactone (PCL) [7], poly(vinyl alcohol) (PVA) [8], cellulose [9], and chitosan (CS) [10]. Among them, CS is the only cationic natural polysaccharide. It is derived from the exoskeleton of crustaceans and from certain fungi. It is a biocompatible, biodegradable, and non-toxic material extensively applied in pharmaceutical and cosmetic applications [11,12,13].

Depending on the desired application, the amelioration of CS properties is feasible. The derivatization of CS and preparation of CS blends are key techniques for improvement in CS innate properties [14]. Through CS derivatization, new characteristic groups are inserted in the CS backbone, ameliorating its solubility or swelling properties. Additionally, the preparation of blends by blending solvents results in the formation of gels with increased viscosity values, an important factor in the 3D printing technique [15].

Atopic dermatitis (AD), also known as atopic eczema, is a chronic inflammatory skin disorder affecting millions of people, particularly infants and young children [16]. It is characterized by pruritic, erythematous, and scaly skin lesions mainly to the flexural surfaces of the body. It is caused by a genetic defect in the filaggrin protein provoking increased skin permeability that incites further itching, scratching, and inflammation [17]. Unfortunately, AD is not curable, and many patients will experience a chronic course of the disease.

The management of AD aims to minimize the exacerbations and alleviate scaly skin [18]. Cream ointments and lotions are utilized to treat AD with topical corticosteroids [19], focusing on increasing skin moisture and protection from bacterial infections corticosteroids [19]. Among the corticosteroids applied for AD treatment, fluticasone propionate (FLU), a synthetic corticosteroid with potent anti-inflammatory properties, has been vastly employed to alleviate patient symptoms. It is best known as an inhaled preparation in asthma and COPD treatment [20]. Nevertheless, topical FLU preparations have been formulated for use in AD. Indeed, FLU, as cream or ointment, underwent in vivo studies in double-blind randomized groups conducted in 2003 [21] and 2017 [22], with exceptional results. Nevertheless, prolonged delivery is necessary for patients’ compliance.

Hence, the aim of the present study was to evaluate the inks designed for 3D printing applications consisting of blends between CS and Vanillin–CS (VACS) derivatives. The preparation of CS/VACS blends was chosen since (i) CS and VACS present high synergic interactions and (ii) inks with high viscosity and printing accuracy were prepared. An examination of the 3D-printed structures and their further use for the preparation of drug-loaded patches was conducted. The effect of the CS/VACS blend ratio on the final 3D-printed structure, as well as the load of FLU on the 3D-printed patches for dermal applications, were examined for the first time.

## 2. Materials and Methods

### 2.1. Materials

Chitosan with high molecular weight (310,000–375,000 Da) and a degree of deacetylation >75% was supplied from Sigma Aldrich Co. (St. Louis, MO, USA). Vanillin with 99% purification was supplied from Alfa Aesar (Thermo Fisher (Kandel) GmbH, Kandel, Germany). Fluticasone propionate (99.99% purity) was kindly donated by Medicair Bioscience S.A. (Athens, Greece). All the other reagents utilized were of analytical grade.

### 2.2. Preparation of VACS, CS/VACS/iCR Inks, and Patches

Vanillin–CS derivative (VACS) synthesis was performed according to our previous paper [23]. Briefly, a 1.6% *v*/*w* CS solution was prepared in an acetic acid solution (1% *v*/*v*), and an ethanolic solution of vanillin (15% *w*/*v*) was added dropwise under magnetic stirring (molar ratio CS/vanillin 1/1.3). The mixture was stirred for 12 h, dialyzed against distilled water for two days, and freeze-dried at −60 °C.

The CS/VACS/iCR inks were prepared at the weight ratios of 70/30, 60/40, and 50/50, according to our previous paper [23], with a few alternations. Briefly, CS and VACS solutions were separately prepared in 2% *v*/*v* acetic acid, and iCR was prepared in a 1% *w*/*v* aqueous solution under heating (80 °C). The solutions were blended under stirring, forming gels with concentrations of 3% *w*/*v*, and iCR was added in a concentration of 1% *w*/*w*. The obtained gels were heated (60 °C) under magnetic stirring in order to evaporate half of the solvent, and the concentrated inks with a final concentration of 6% *w*/*v* were acquired.

The blends were pneumatically extruded using an extrusion-based 3D Bioprinter (CELLINK^®^ Inkredible, Gothenburg, Sweden), through a nozzle with an inner diameter of 0.6 mm (G20). An STL file of a three-dimensional rectangle (3 × 3 × 0.6 cm) was utilized for 3D printing, while the slicing of the STL sample was performed with the Slic3r software (infill 80%, 19 layers, angle of layers 0°). The printing conditions were individually optimized for each sample, and they are summarized in Table 1. The post-printing crosslinking of the obtained structures was conducted with 12% *w*/*v* sodium hydroxide (NaOH) in a 30% *v*/*v* ethanol (EtOH) solution for 60 min. After that, the samples were washed several times with a H_2_O/EtOH 50/50 *v*/*v* solution, suspended in deionized water, frozen, and freeze-dried. All the printed samples were depicted through a stereomicroscope (SteREO Discovery V.20, Zeiss, Germany) equipped with a camera (GRYPHAX Altair, Jenoptik, Jena, Germany). The scaffolds’ line diameters and pore sizes were measured using stereomicroscope images.

The drug-loaded samples were prepared through absorption according to the following procedure: The accurately weighted amounts of FLU, corresponding to 5, 10, and 20 wt% of FLU to the dry scaffold, were dissolved in a mixture of H_2_O/methanol (50/50 *v*/*v*). Then, the freeze-dried samples were inserted into the prepared drug solution and left until the drug was completely absorbed. The resultant FLU-loaded samples were frozen and freeze-dried under reduced pressure at −60 °C in order to obtain the final FLU-loaded samples. Each sample was printed multiple times for various physicochemical measurements.

### 2.3. Viscosity Measurements

The viscosity measurements of the inks were performed at three different temperatures, 25 °C, 40 °C, and 55 °C, together with increasing the rotational speed (20–60 rpm) using the SC29 spindle of a rheometer (BGD 157/TS, Biuged Instruments, Guangzhou, China).

### 2.4. Characterization of 3D-Printed Structures

#### 2.4.1. Fourier Transformed Infrared Spectroscopy (FTIR)

The FTIR spectra of the samples were obtained using an FTIR spectrometer (model FTIR-2000, PerkinElmer, Waltham, MA, USA). In brief, a small amount of each sample was triturated with a proper amount of potassium bromide (KBr), and the disks were formed under pressure. The spectra were collected in the range from 400 to 4000 cm^−1^ at a resolution of 4 cm^−1^ using 16 coadded scans, while the baseline was corrected and converted into absorbance mode.

#### 2.4.2. Wide-Angle X-ray Scattering (XRD)

The X-ray diffraction (XRD) patterns were reported using an XRD diffractometer (Rigaku-Miniflex 600, Chalgrove, Oxford, UK), with CuKα radiation for crystalline phase identification (λ = 0.15405 nm). All the samples were scanned with 2θ ranging from 5° to 50° and a scan speed of 1°/min.

#### 2.4.3. Contact Angle

For the calculation of the contact angle, films of approximately 2 × 2 cm^2^, prepared via the solvent evaporation of 1% *w*/*v* polymeric solutions at 50 °C, were placed onto a microscope glass. The contact angles were measured in water, employing the sessile drop method with Ossila Contact Angle Goniometer L2004A1 (Ossila Ltd., Sheffield, UK). The experiment was performed in triplicate. The results are expressed as mean ± standard deviation (SD).

#### 2.4.4. Swelling and Water Content Capacity

The swelling ability of the prepared printed patches was evaluated by measuring the amount of the water sorption aptitude of a simulated body fluid (SBF) buffer (pH = 7.4). Each dry sample was carefully weighed (Wd) and immersed in SBF. The samples were then placed on filter paper in order to remove the excess surface water, and their weight (Wf) was measured at predetermined times (5 min, 10 min, 20 min, 30 min, 1 h, 2 h, 3 h, and 48 h). The swelling ratio and water content were calculated according to Equations (1) and (2), respectively.

Swelling ratio% = (W_f_ − W_d_) × 100/W_d_ (1)

Water content% = (W_f_ − W_d_) × 100/W_f_ (2)

The dehydration progress of the samples was evaluated by measuring the water content loss of the samples. The samples were placed in water for 24 h (W_0_, water content 100%) and then placed in a vacuum oven (40 °C, 200 mbar). The weight of the samples (W_f_) was measured at predetermined times (5 min, 10 min, 20 min, 30 min, and 60 min). The measured weight was compared with the initial weight of the dry samples (W_d_, water content 0%). The relative water content was assessed through Equation (3). The measurements were performed in triplicate.

Relative water content = (W_f_ − W_d_) × 100/W_0_ − W_d_ (3)

#### 2.4.5. Enzymatic Hydrolysis

The enzymatic hydrolysis of the samples was evaluated by placing the samples in 5 mL of SBF, pH = 7.4, containing 1 mL of a lysozyme solution (0.8 mg/mL). The samples were then placed in an oven at 37 °C and at predetermined times (0 h, 24 h, 48 h, 72 h, 96 h, 144 h, and 240 h); they were washed with distilled water, vacuum-dried in an oven at 50 °C, and weighed. The measurements were performed in triplicate.

#### 2.4.6. High-Pressure Liquid Chromatography (HPLC), Quantitative Analysis and Drug Loading Quantitative

Quantitative analysis and drug loading were conducted utilizing a Shimadzu HPLC (Kyoto, Japan) system consisting of a degasser (DGU-20A5, Kyoto, Japan), a liquid chromatograph (LC-20 AD, Kyoto, Japan), an autosampler (SIL-20AC, Kyoto, Japan), a UV/Vis detector (SPD-20A, Kyoto, Japan), and a column oven (CTO-20AC, Kyoto, Japan). The samples were eluted with an isocratic method described by Jetzer et al. [24]. The column was a type of CNW Technologies Athena C18, 120 A, 5 µm, 250 mm × 4.6 mm set at room temperature. Briefly, the mobile phase consisted of ACN/H2O/TFA (58/42/0.1 *v*/*v*/*v*), and FLU was detected at the wavelength of 238 nm. The flow rate through the HPLC system was 1 mL/min, the adjusted injection volume was 10 µL, while the sharp peaks were obtained at approximately 14 min. The calibration curve was developed by diluting a 500 ppm stock methanol solution of the drug to the concentrations of 0.01, 0.05, 0.1, 0.25, 0.5, 1.0, 2.5, 5.0 10.0, 20.0, 30.0 and 50.0 ppm, using ultrapure water. For further demonstration of the drug-loading capacity of the 3D-printed patches, 10 mg printed patches were dissolved in 10 mL of aqueous acetic acid solution (1% *v*/*v*): methanol (50/50 *v*/*v*). The subsequent solution was stirred for 24 h and filtered through nylon filters (0.45 nm pore size). The percentage of drug loading was calculated using the following equation:

Drug loading (%) = [Weight of drug in patches/Weight of patches] × 100(4)

#### 2.4.7. In Vitro Dissolution Studies

In vitro release studies were performed with the aid of DISTEK Dissolution Apparatus using the paddle method (USP II) (North Brunswick, NJ, USA) equipped with an autosampler (Evolution 4300, North Brunswick Township, NJ, USA). The 3D-printed patches were placed into dissolution vessels corresponding to 75 mg of each formulation in an appropriate patch holder, with its application side up. The dissolution operation was performed at 32 ± 1 °C, with a rotation speed of 100 rpm. The dissolution medium was 500 mL of a phosphate buffer adjusted at pH = 7.4. At predefined time intervals, 2 mL of the aqueous solution was withdrawn from the release media and further analyzed for the actual drug content using HPLC, as previously described.

## 3. Results and Discussion

### 3.1. Characterization of CS/VACS Inks

**Figure 1:**
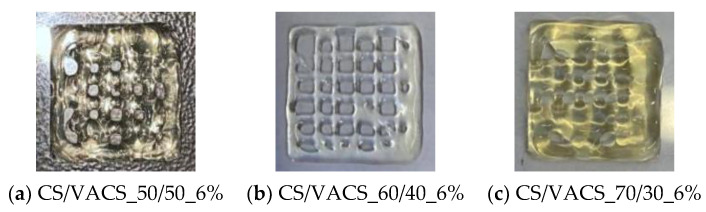
The 3D-printed multilayered scaffolds of CS/VACS 6% *w*/*v* (**a**) 50/50, (**b**) 60/40, and (**c**) 70/30.

In our previous paper, inks consisting of CS/VACS 5 and 6% *w*/*w* blends were promising candidates for a successful pneumatic extrusion since they were able to extrude uniformly. However, when printing multiple layers, the inks were not able to maintain their structure and collapsed under the gravity force (Figure 1). Consequently, the addition of a gelling agent in order to maintain the dimensional accuracy of the printed samples is mandatory. ι-carrageenan (iCR) is a natural polysaccharide, with exceptional characteristics as a gelling agent since it forms elastic gels stable through refrigerated storage, freezing, and thawing procedures [25]. Therefore, 1% *w*/*w* iCR was applied as a thickening agent in the CS/VACS blends.

The preparation of inks with certain printable behavior greatly depends on the flow ability of the bioinks. In general, an ideal ink for 3D printing should present a shear thinning behavior [26]. The utilized materials ought to be sufficiently viscous to retain their shape during the printing process but not too viscous, in order to obtain uniform strands and avoid nozzle clogging. Ink’s high viscosity value is beneficial during 3D printing as well as during post-printing since the prepared filament is firm and difficult to flow and spread. Therefore, the shape of both the filament and the final sample is retained [27]. The inks with viscosity values exceeding 10.000 Pa·s are characterized as too viscous; when their viscosity is lower than 100 Pa·s, they are characterized as too fluidic [23]. CS has been applied in many 3D printing applications owing to its innate nature of forming non-Newtonian gels with high viscosity values, which is an essential requirement for a successful 3D printing application [2].

**Figure 2:**
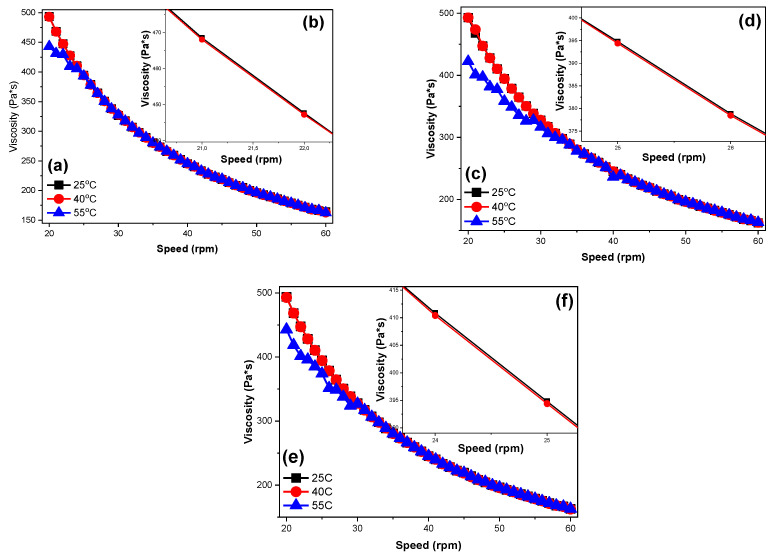
Viscosity dependency over increasing rpm values of the samples: (**a**) CS/VACS/iCR 50/50 20–60 rpm, (**b**) CS/VACS/iCR 50/50 21–22 rpm, (**c**) CS/VACS/iCR 60/40 20–60 rpm, (**d**) CS/VACS/iCR 60/40 25–26 rpm, (**e**) CS/VACS/iCR 70/30 20–60 rpm, and (**f**) CS/VACS/iCR 70/30 24–25 rpm.

The viscosity of the new-formed CS/VACS/iCR inks was assessed through rheological measurements via increasing the rotational speed. The viscosity values of the CS/VACS/iCR blends with increasing rpm are presented in Figure 2. As depicted, the blends presented a shear-thinning behavior with a decrease in viscosity values as the rotational speed increased. It is interesting that the viscosity values of the blends were slightly affected by the application of different temperatures. Typically, in CS gels, an increase in the applied temperature results in a decrease in its viscosity value [28]. As depicted in Figure 2b,d,f, the increase in temperature at 40 °C had a small impact on the viscosity values of the blends. A further increase in the temperature to 55 °C affected the viscosity values to a higher extent. In the CS/VACS/iCR 50/50 ink, the only deviation was observed in the low rotational speed (20–25 rpm), while in the samples CS/VACS/iCR 60/40 and CS/VACS/iCR 70/30, a decrease in the viscosity values was detected during the initial 12 rpm (20–32 rpm) and 9 rpm values (20–29 rpm), respectively. These steady viscosity values are attributed to the extensive hydrogen bonds formed between CS, VACS, and iCR. Nevertheless, the viscosity values of all the samples lay within the appropriate values and rendered the CS/VACS/iCR samples exceptional candidates for their further 3D printing.

### 3.2. Characterization of 3D-Printed Structures

#### 3.2.1. Morphological Characterization

**Figure 3:**
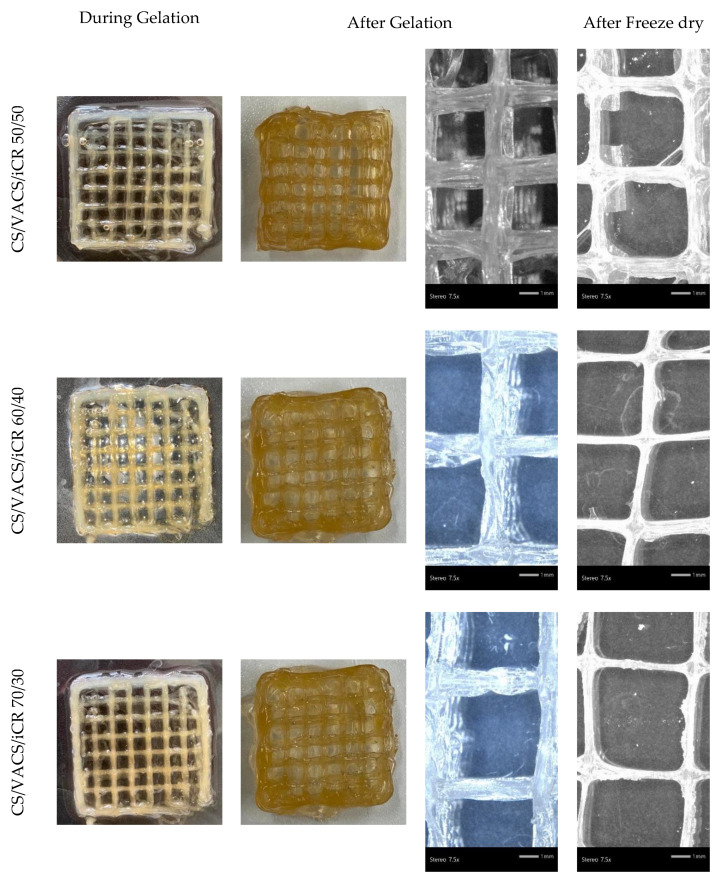
Images of the samples CS/VACS/iCR 50/50, 60/40, and 70/30 before and after gelation and stereomicroscope images of the samples after gelation and after freeze-drying.

During 3D printing, the ability to control the resolution and dimensionality of the scheme is a key point for the successful preparation of 3D constructs [29]. The macroscopic images of the CS/VACS/iCR constructs are presented in Figure 3 in the three different ratios analyzed. In any ratio, squared grid structures with excellent shape fidelity were successfully prepared. Their dimensional stability before and after gelation was evident since the void area of the grids was unaffected. The stereoscopy images before and after the drying procedure are also presented in Figure 3. The preparation of multilayered scaffolds with distanced lines and clear squared grids was apparent.

The addition of a strong NaOH solution to the printed structures leads to the augmentation of the pH value reaching the alkali region, where both CS and VACS are insoluble. The increase in the pH above 6.5 leads to a sol–gel transition of CS and VACS, forming a three-dimensional network due to the generation of physical junctions of H bonds [30]. The primary amino groups are neutralized, forming extensive hydrophobic interactions and hydrogen bonds among the different moieties present in the CS and VACS backbones, such as amines, hydroxyl, and carbonyl groups [31].

**Figure 4:**
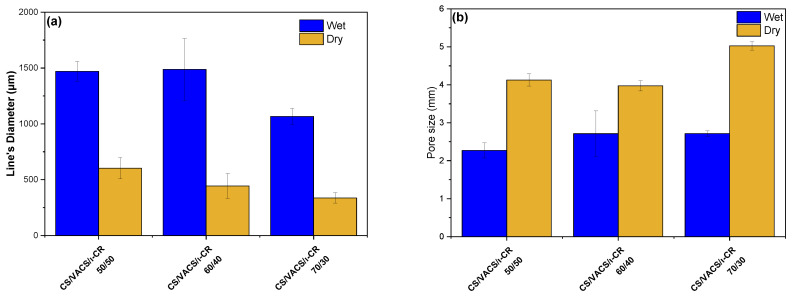
Average (**a**) scaffold’s line diameter and (**b**) pore sizes of the samples CS/VACS/iCR 50/50, 60/40, and 70/30. Five measurements were performed for each sample.

In the further step of the process, the drying of the scaffolds is important since their storage, transfer, and study are easier in comparison to wet scaffolds [32]. After the freeze-drying process, the pores’ sizes were enlarged, while the scaffolds’ lines were smaller. Figure 4a,b present the average scaffold’s line diameters and pore sizes, respectively. Regarding the pore size, the scaffolds with larger diameters naturally presented a smaller pore size and vice versa. As can be observed, when the scaffolds contained a solvent, their pores had a smaller diameter, while their grid line diameters were wider. After the drying procedure, their pores were enlarged, while the grid lines were narrower. It is interesting that, as the percentage of the VACS increased, the average line was augmented. This observation is probably attributed to the extended hydrogen bonds between CS and VACS, leading to more swelled scaffolds with wider lines and narrower pores. After the removal of the solvent, the dry constructs appeared to have wider pores and narrower lines.

#### 3.2.2. Physicochemical Characterization

**Figure 5:**
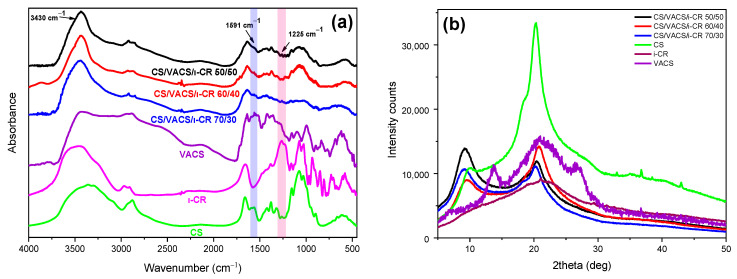
(**a**) FTIR spectra and (**b**) X-ray spectra of the samples CS, VACS, iCR, and CS/VACS/iCR with 50/50, 60/40, and 70/30 ratios.

The successful preparation of the VACS derivative as well as the interaction between neat CS and VACS initial materials were well-established in our previous paper through FTIR measurements [23]. In brief, CS characteristic peaks were present at 3000–3600 cm^−1^, which are attributed to the broad overlapped peak of O–H and N–H bonds. The bands at 1651 cm^−1^ and 1567 cm^−1^ are attributed to amide I and II, respectively, while further peaks were present at 1421 (C–H and O–H vibrations), 1326 (C–N axial deformation), 1154 (anti-symmetric stretching of the C–O–C bridge) and 1077 cm^−1^ (skeletal vibrations, C–O stretching) [33]. Concerning the VACS derivative, the characteristic peaks of CS were present in the VACS spectra; however, they were slightly shifted to lower wavenumbers. Moreover, the characteristic C–N peak was present at 1591 cm^−1^, confirming successful derivatization. Subsequently, the CS/VACS/iCR samples were examined through FTIR spectra (Figure 5a). The overlapped curve attributed to the hydrogen bonds of hydroxyl and amino groups present in the CS and VACS structure was detected in all the CS/VACS/iCR ratios. Due to the physical gelation of the samples, the overlapped peak was narrower [34]. Moreover, amides I and II were not clearly distinguished in the CS/VACS/iCR samples, due to the gelation of the samples with the NaOH solution. Nevertheless, the characteristic peak of the VACS derivative, present at 1591 cm^−1^ and indicative of the C–N bond, increased by increasing the VACS ratio. Finally, the iCR was hardly distinguishable in the FTIR spectra owing to its low concentration, i.e., 1% *w*/*w*, while its characteristic peaks overlapped with the CS or VACS peaks. However, the peak present at 1225 cm^−1^ is attributed to the ester sulfate of the iCR, confirming the presence of iCR in the samples [35].

In a further step, the CS/VACS/iCR gelated samples were examined through XRD measurements (Figure 5b). CS is a semicrystalline polysaccharide with two characteristic peaks at 10 and 20°, while the semicrystalline behavior of the VACS derivative was described in detail in our previous work, with three distinct peaks at 13.8°, 21°, and 26.9° [23]. The CS/VACS/iCR samples presented a slightly modified behavior in comparison to those of the initial materials. The peak of CS at 20.2° shifted to 20.8° in the 60/40 ratio, which is attributed to the VACS ratio of the blends, while its intensity diminished. Furthermore, the peak of CS at 10° shifted to 9°, while its intensity varied according to the content of VACS. The gelation of the samples with NaOH is critical for their crystalline structure. The sol–gel transition occurring during gelation provokes a rearrangement of the polymeric chains, leading to changes in the crystalline structure of the CS/VACS/iCR printed structures. This behavior is described in the literature with the study of Takara et al., who observed the differentiation in CS semicrystalline peaks when treated with different NaOH solutions [36].

**Figure 6:**
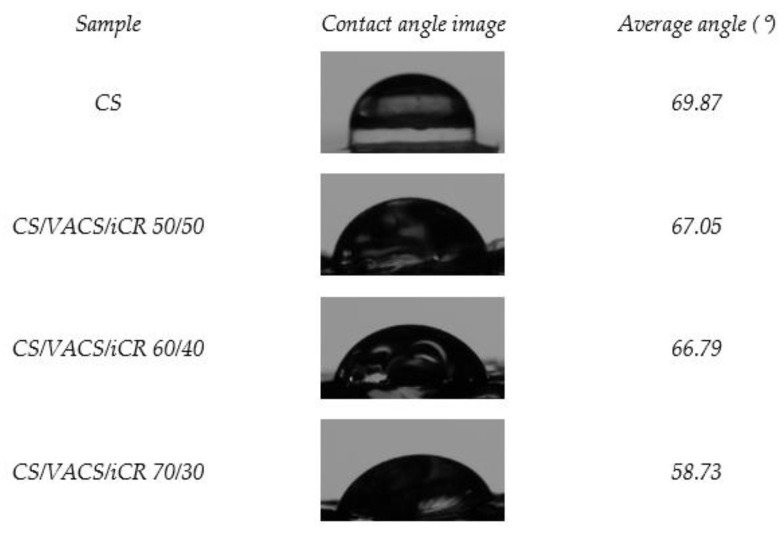
Contact angle images and their values of the CS/VACS/iCR 50/50, 60/40, and 70/30.

In the pharmaceutical field, the wettability of the utilized materials is an important factor in describing the interaction between the surface of a tablet or patch with the body fluids. The absorption rate of the liquid into the surface has an impact on the drug release in a particular dissolution medium [37]. More specifically, when designing skin patches for atopic skin, their innate wetting properties are important [38] since transepidermal water loss is one of the abnormalities of atopic skin. Consequently, those materials able to reduce water vapor are preferable [39]. The contact angle is a relatively simple method, commonly used for estimating the hydrophilicity or hydrophobicity of a surface through the measurement of the angle made in a solid/liquid/gas interface on a surface [40]. Those materials with values θ < 90° are described as hydrophilic, while with θ values of 90° < θ < 150° or θ > 150°, the surface of the materials is characterized as hydrophobic and super-hydrophobic, respectively [37]. Figure 6 presents the contact angle measurements of neat CS and the samples CS/VACS/iCR 50/50, 60/40, and 70/30. Neat CS had a value of 69.87°, and its high contact angle value is attributed to the hydrophobic backbone of its chains. When examining the CS/VACS/iCR films, the decreased contact angle values are attributed to the higher hydrophilicity of the VACS derivative. These results are in accordance with the literature data where the water content of CS/xylan and CS/cellulose blends were examined [41,42]. Furthermore, it is evident that by increasing the ratio of VACS, the average θ value increased, probably attributed to the increased presence of the phenolic ring of the vanillin monomer on the VACS backbone.

**Figure 7:**
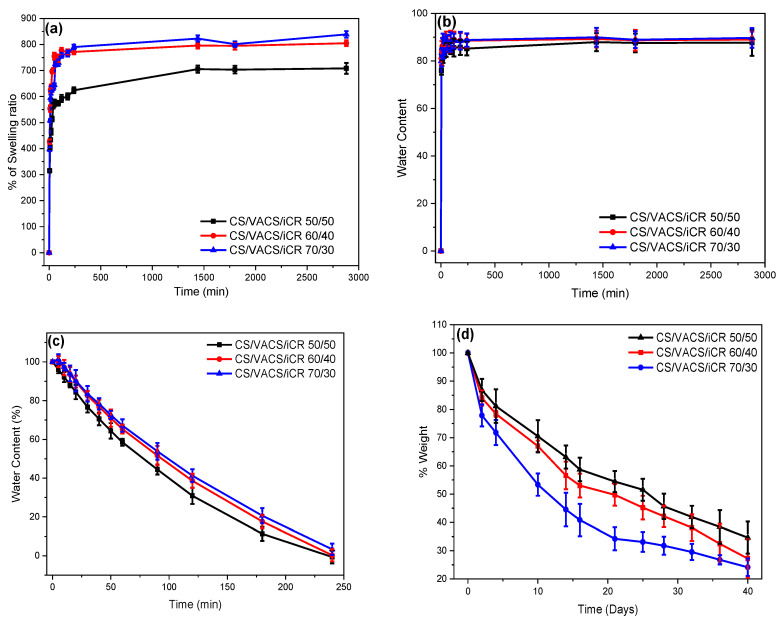
(**a**) Degree of swelling, (**b**) water content, (**c**) dehydration, and (**d**) enzymatic hydrolysis of the samples CS/VACS/iCR 50/50, 60/40, and 70/30 as a function of time.

One significant characteristic of the polymeric materials designed for dermal patches is their ability to swell in aqueous environments. Naturally, CS is able to swell its dry mass up to 500% *w*/*w*, while its derivatives with hydrophilic monomers are able to swell their dry mass up to 5000% *w*/*w* [20,23,43]. Figure 7a,b present the swelling ability and the water content of the 3D-printed CS/VACS/iCR samples with 50/50, 60/40, and 70/30 ratios, respectively. The swelling ability of the samples lay between 600% and 800% of their dry mass, while higher swelling ability was observed in the sample with the higher CS content. This result is attributed to the presence of the aromatic ring of vanillin in the VACS derivative. The presence of the large ring affected the ability of the blends to swell and retain large amounts of water in their structure owing to steric hindrance. The swelling process follows a two-phase behavior: an initial fast swelling phase during the first 1 h followed by a steady swelling phase. In addition, water content follows a similar pattern to swelling efficiency. The higher water content was observed in the sample CS/VACS/iCR 70/30 with the higher content in CS equal to 89%. The swelling efficiency and water content were lower in the printed samples in comparison to the initial CS and VACS materials, which is attributed to gelation. This observation is in accordance with the literature data where the gelation of hydrogels was revealed to have a great impact on their swelling efficacy [44].

An important factor for hydrogels and drug delivery patches is their dehydration ability or swelling reversibility [45]. Figure 7c presents the dehydration of the swollen samples. The samples presented a reversible behavior in terms of their swelling ability. The fastest dehydration was observed in the sample CS/VACS/iCR 50/50, followed by those with ratios of 60/40 and 70/30. The sample with a 50/50 ratio retained the least amount of water during the swelling process in comparison to those with the ratios of 60/40 and 70/30, resulting in faster dehydration. However, even though during the swelling behavior, rapid retention of water was observed, with a fast swelling step during the first hour, the dehydration occurred gradually, with a linear relationship over time.

In the next step, the enzymatic hydrolysis of the 3D-printed structures was examined (Figure 7d). Hydrolysis provokes the depolymerization of CS and VACS through the scission of the sensitive β-1,4 glycosidic bond, forming aldehyde groups [46]. The mass loss during hydrolysis is defined by various factors, namely polymeric concentration, degree of deacetylation, specific surface area, and swelling behavior [47]. In Figure 7, the swelling-dependent mass loss behavior of the CS/VACS/iCR samples is evident. The sample CS/VACS/iCR 70/30, with the higher swelling ability, reduced its mass up to 24%, whereas the samples with 60/40 and 50/50 ratios presented a reduction in their mass of up to 28% and 34%, respectively. The higher swelling ability resulted in a more efficient distribution of the enzyme into the polymeric chains, leading to a higher degradation of the glycosidic bonds and subsequently diminishing the mass.

#### 3.2.3. Characterization of Drug-Loaded Patches

**Figure 8:**
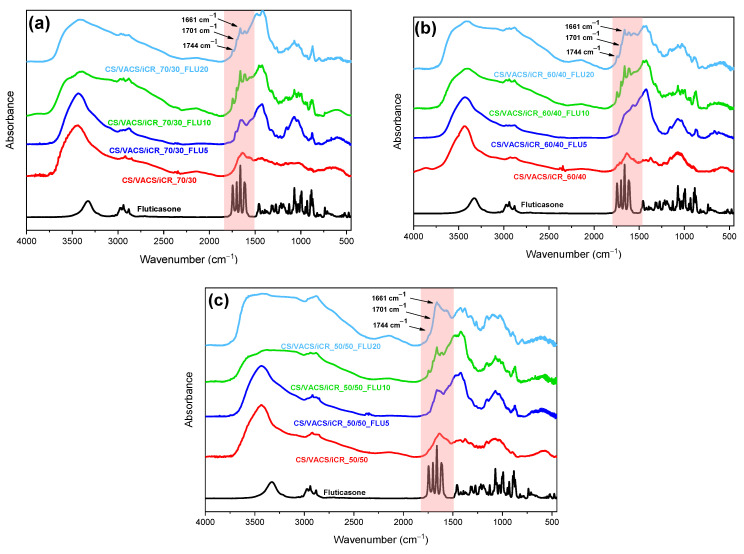
FTIR spectra: (**a**) CS/VACS/iCR 70/30, (**b**) CS/VACS/iCR 60/40, and (**c**) CS/VACS/iCR 50/50 containing FLU in different ratios of 5%, 10% and 20% *w*/*w*.

For the study of the successful loading of FLU in the CS/VACS/iCR samples and the determination of the potential interactions between the drug and polymeric matrixes, FTIR measurements were performed (Figure 8a). Regarding the CS/VACS/iCR spectra, all their characteristic peaks were observed. According to the literature, neat FLU displays its principle sharp peaks at 1744 cm^−1^, corresponding to the ester carbonyl group (C=O); at 1701 cm^−1^, attributed to the thioester carbonyl group; at 1661 cm^−1^, attributed to the ketone groups (C=C); and at 1409 cm^−1^, which is characteristic for the stretch of the hydroxyl group (–OH). Moreover, thiol stretch is detected at 991 cm^−1^ (S–H), fluorine stretches at 1024 cm^−1^ (C–F), and ether groups stretch at 883 cm^−1^ [20,48]. In the FTIR spectra of the FLU-loaded samples, all the FLU characteristic peaks were detected throughout the spectra without any noticeable changes in band position, indicating the absence of interactions between FLU and the polymeric matrix. This result is in agreement with the literature [49]. Nevertheless, the peaks corresponding to ester carbonyl groups, thioester carbonyl groups, and ketone groups at 1744 cm^−1^, 1701 cm^−1^, and 1661 cm^−1^, respectively, were present in the CS/VACS/iCR samples, indicating that FLU was successfully loaded.

**Figure 9:**
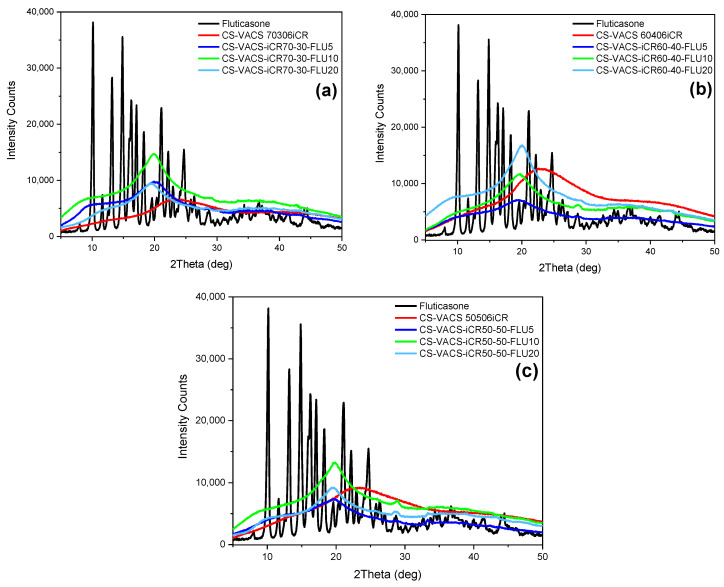
XRD diffractograms: (**a**) CS/VACS/iCR 70/30, (**b**) CS/VACS/iCR 60/40, and (**c**) CS/VACS/iCR 50/50, containing FLU in different ratios 5%, 10% and 20% *w*/*w*.

In the next step, XRD analysis was performed to determine the physical state of the FLU loaded into CS/VACS/iCR structures. In general, the crystallinity of active compounds has a great impact on the properties of the final products. The solubility and dissolution rate are the most important factors [50]. According to a previous study of our group, higher crystallinity results in a lower dissolution rate [51]. Consequently, the loading of FLU should occur in its amorphous state. Figure 9b presents the outcomes of the XRD measurements. FLU is a highly crystalline compound, with characteristic peaks throughout its diffractogram [52]. Contrarily, the CS/VACS/iCR 3D-printed samples were amorphous in all the ratios under study, with a characteristic hollow shape. In the drug-loaded patches, FLU was loaded in its amorphous phase since no crystalline peak was detected in the diffractograms from any of the ratios. DSC measurements were performed to confirm the XRD results (Figure S1). FLU, as a crystalline compound, presents a melting peak at 300 °C [53]. According to the literature, CS is a polysaccharide with a characteristic endothermic peak at 60–100 °C, ascribed to its loss of moisture and exothermic peak at 295 °C, when the decomposition of the polymeric matrix occurs [54]. None of the ratios of the CS/VACS/iCR-FLU samples revealed the characteristic endothermic peak of FLU while the decomposition of polysaccharides occurred. These results confirmed the amorphous loading of FLU into the 3D-printed structures.

The loading efficacy of the patches is a characteristic drastically affecting their further in vitro release behavior, leading to the optimum absorption, and consequently relief, of the patient [55]. Table 2 summarizes the drug-loading efficacy of all the patches. Our results indicated that efficient drug loading was achieved in all the CS/VACS ratios for the patches theoretically containing FLU 20 wt%. The actual percentages were 14.3%, 19.4%, and 19.1% for 50/50, 60/40, and 70/30 ratios, respectively. Concerning the samples containing FLU 10 wt%, their actual drug-loading efficacy was 2%, 5.7%, and 9.7% for the CS/VACS samples with ratios of 50/50, 60/40, and 70/30, respectively. Regarding the samples containing FLU 5 wt%, their drug-loading efficacy was negligible. It is interesting that concerning the CS/VACS sample with a 50/50 ratio, the drug-loading efficacy was lower, probably due to the steric hindrance effects of the aromatic ring of the vanillin present in the VACS derivative.

**Figure 10:**
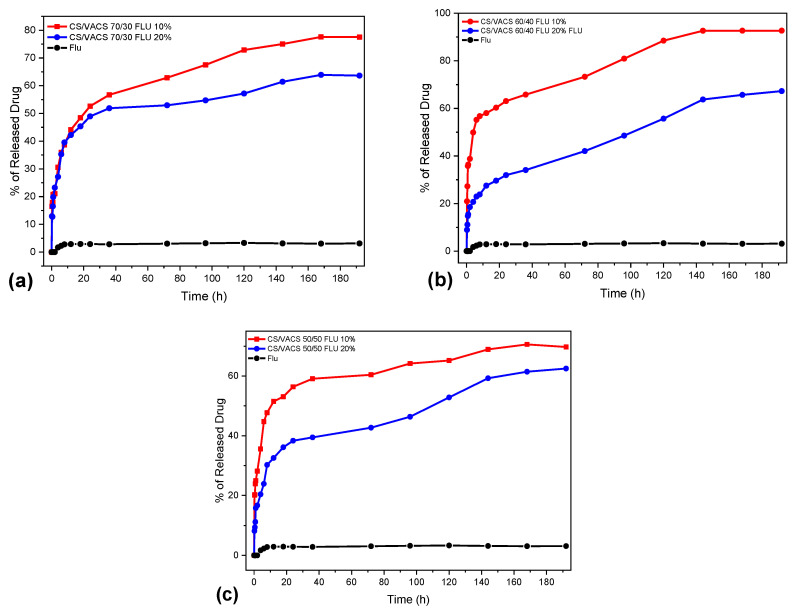
In vitro release of FLU from CS/VACS/iCR patches at pH 7.4. (**a**) CS/VACS 70/30, (**b**) CS/VACS 60/40, (**c**) CS/VACS 50/50.

In vitro dissolution studies were also conducted for the evaluation of FLU release rate. Figure 10 shows the dissolution profiles of neat FLU and CS/VACS/iCR patches. Since the drug loading in the ratios containing FLU 5 wt% was very low, their further in vitro dissolution profiles were not examined. FLU, as a glucocorticosteroid with low aqueous solubility and high hydrophobicity, exhibits a low dissolution profile [56]. As shown in Figure 10, neat FLU practically remained undissolved, while its dissolution profile did not exceed 4% after 8 days.

Concerning the release of FLU from the 3D-printed patches, our dissolution studies revealed an optimization of its in vitro behavior since all the blends displayed a significant increase in the amount of the dissolved FLU in comparison to neat FLU. The improvement in the drug’s dissolution rate from the patches is attributed to FLU loading into the patches in the amorphous phase. According to Teja et al. [57], the amorphous phase of the crystal compounds has higher solubility and an enhanced dissolution rate compared with the crystal phase. The release of FLU from the patches followed a two-phase release profile. An initial burst release was detected during the first 10 h, followed by a sustained release up to 8 days. The samples containing FLU 20 wt% achieved a release of up to 62%, 67%, and 63% for the CS/VACS ratios 50/50, 60/40, and 70/30, respectively, whereas when containing FLU 10 wt%, the maximum release was 70%, 93%, and 77% for the CS/VACS ratios 50/50, 60/40, and 70/30, respectively. Noteworthy is the fact that, by increasing the percentage of the FLU present in the patches, the in vitro release ability decreased. This inverted correlation between drug content and drug release behavior is typical for poorly water-soluble drugs and is attributed to the formation of hydrophobic interactions among the molecules of the drugs [51,58].

In addition, the release behavior of drugs in terms of swelling matrixes is heavily dependent on their innate swelling ability. In this context, the release profiles are associated with the ratios of the CV/VACS blends. The ratio with the higher content of VACS resulted in the lowest swelling ability and, consequently, decreased dissolution ability. By contrast, according to the swelling data, the optimum dissolution was expected from the sample CS/VACS/iCR 70/30 FLU 10%. However, complete dissolution ability was observed in the sample CS/VACS/iCR 60/40 FLU 10%. The main reason for this behavior is that other than swelling: drug dissolution and diffusion along with the erosion of the polymers also contribute to this result [59].

These in vitro dissolution results render the CS/VACS/iCR FLU-loaded samples promising candidates for the treatment of AD. However, further studies are required on in vitro keratinocytes and fibroblast cell cultures [60] for confirming the biocompatibility and suitability of the patches analyzed in this study. Moreover, in vivo animal models [61] would support the applicability of these patches for the alleviation of AD symptoms.

## 4. Conclusions

In the present study, CS/VACS/iCR blends were prepared for the formation of 3D-printed patches for the administration of FLU. The CS/VACS/iCR blends revealed appropriate viscosity values for extrusion with a 3D printer. The 3D-printed samples were able to maintain their shape after printing and crosslinking procedures, while their swelling ability and hydrophilicity were found to be dependent on the CS/VACS ratio, providing higher swelling ability and hydrophilicity in the samples with the lowest ratio of VACS. Furthermore, FTIR measurements confirmed the successful loading of FLU into the 3D-printed samples, while XRD and DSC measurements established the amorphous state of FLU. Finally, in vitro release studies indicated the enhanced release of FLU in the dissolution medium, while the complete release of FLU was obtained from the sample CS/VACS/iCR 60/40 FLU 10%. The aforementioned results render the 3D-printed CS/VACS/iCR FLU samples potential candidates for the treatment of AD.

## Figures and Tables

**Table 1:** Optimum printing parameters of the CS/VACS/iCR inks.

Sample	Infill (%)	Speed (mm/s)	Pressure (kPa)	Temperature
CS/VACS/iCR 70/30	80	8	200	RT
CS/VACS/iCR 60/40	80	8	220	RT
CS/VACS/iCR 50/50	80	8	240	RT

**Table 2:** FLU loading percentage in CS/VACS/iCR patches.

Sample		Drug Loading (%)
CS/VACS/iCR 50/50	FLU 5%	0.7
	FLU 10%	2.0
	FLU 20%	14.3
CS/VACS/iCR 60/40	FLU 5%	0.4
	FLU 10%	5.7
	FLU 20%	19.4
CS/VACS/iCR 70/30	FLU 5%	0.6
	FLU 10%	9.7
	FLU 20%	19.1

## Data Availability

Data are contained within the article.

## Supplementary Materials

The following supporting information can be downloaded at: https://www.mdpi.com/article/10.3390/pharmaceutics14091966/s1, Figure S1: DSC curves of CS/VACS/iCR samples containing FLU in 5, 10, and 20 wt%.
