# Supplementary figures and images for: Novel 3D-Printed Dressings of Chitosan–Vanillin-Modified Chitosan Blends Loaded with Fluticasone Propionate for Treatment of Atopic Dermatitis
Source: Pharmaceutics. 2022 Sep 18;14(9):1966. doi: 10.3390/pharmaceutics14091966 (PMC9503579; doi:10.3390/pharmaceutics14091966)

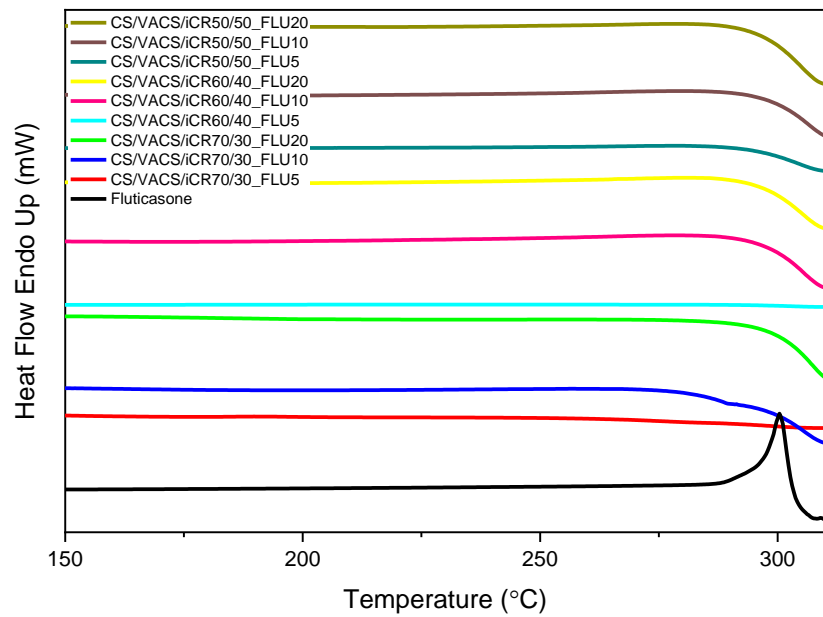

**Figure S1.** DSC curves of CS/VACS/iCR samples containing FLU in 5, 10 and 20 wt%.

Supplement: Supplementary file 1 [file pharmaceutics-14-01966-s001.zip › pharmaceutics-1919666-supplementary.pdf]
